# Supplementary material for: Extended-spectrum beta-lactamase-producing Escherichia coli and Klebsiella pneumoniae from human carriage, the human-polluted environment, and food: Molecular epidemiology of two prospective cohorts in five European metropolitan areas
Source: PLoS One. 2026 Jan 7;21(1):e0337346. doi: 10.1371/journal.pone.0337346 (PMC12779062; doi:10.1371/journal.pone.0337346)
Supplement: Attachment — Submitted filename: Response to Reviewers.docx [file pone.0337346.s004.docx]

The Editorial Team

PLOS One

1875 Mission Street

Suite 103 #188 San Francisco, CA 94103

United States

10 October 2025

Dear PLOS One Editors,

We would like to sincerely thank the editors and reviewers for taking the time to review and provide valuable commentary on our manuscript. Below, we provide a point-by-point response, with each comment followed by our response marked in yellow.

With kind regards,

Tess Verschuuren

Corresponding author

t.d.verschuuren@gmail.com

**Journal Requirements:**

1. Please ensure that your manuscript meets PLOS ONE's style requirements, including those for file naming. The PLOS ONE style templates can be found at https://journals.plos.org/plosone/s/file?id=wjVg/PLOSOne_formatting_sample_main_body.pdf and https://journals.plos.org/plosone/s/file?id=ba62/PLOSOne_formatting_sample_title_authors_affiliations.pdf

We adjusted the manuscript based on PLOS ONE’s style requirements. Including font, lay-out of headers, from British English to American English, and reference style.

2. Thank you for stating in your Funding Statement:

[The MODERN studies (Understanding and modelling reservoirs, vehicles and transmission of ESBL-producing Enterobacteriaceae in the community and long term care facilities) were part of a Joint Programming Initiative on Antimicrobial Resistance collaborative research project, under the 2016 Joint Call framework (Transnational Research Projects on the Transmission Dynamics of Antibacterial Resistance). It received funding from the following national research agencies: Instituto de Salud Carlos III (grant no. AC16/00076), Netherlands Organization for Health Research and Development (grant no. 681055, 547001004), Swiss National Science Foundation (grant no. 40AR40-173608), German Federal Ministry of Education and Research (grant no. 01KI1701), the French Agence Nationale de la Recherche (grant no. ANR-16-JPEC-0007-03), and UK Medical Research Council (grant no. MR/R004536/1). Elena Salamanca, and Jesús Rodríguez-Baño received support for research from by the Plan Nacional de I+D+i 2013-2016 and Instituto de Salud Carlos III, Subdirección General de Redes y Centros de Investigación Cooperativa, Ministerio de Ciencia, Innovación y Universidades, Spanish Network for Research in Infectious Diseases (REIPI RD16/0016/0001), co-financed by the European Development Regional Fund A way to achieve Europe, Operative Program Intelligence Growth 2014–2020.].

Please provide an amended statement that declares *all* the funding or sources of support (whether external or internal to your organization) received during this study, as detailed online in our guide for authors at http://journals.plos.org/plosone/s/submit-now. Please also include the statement “There was no additional external funding received for this study.” in your updated Funding Statement.

Please include your amended Funding Statement within your cover letter. We will change the online submission form on your behalf.

We have amended the funding statement, and included ‘there was no additional external funding received for this study, see below.
**Funding statement**
The MODERN studies (Understanding and modelling reservoirs, vehicles and transmission of ESBL-producing Enterobacteriaceae in the community and long term care facilities) were part of a Joint Programming Initiative on Antimicrobial Resistance collaborative research project, under the 2016 Joint Call framework (Transnational Research Projects on the Transmission Dynamics of Antibacterial Resistance). It received funding from the following national research agencies: Instituto de Salud Carlos III (grant no. AC16/00076), Netherlands Organization for Health Research and Development (grant no. 681055, 547001004), Swiss National Science Foundation (grant no. 40AR40-173608), German Federal Ministry of Education and Research (grant no. 01KI1701), the French Agence Nationale de la Recherche (grant no. ANR-16-JPEC-0007-03), and UK Medical Research Council (grant no. MR/R004536/1). Elena Salamanca, and Jesús Rodríguez-Baño received support for research from by the Plan Nacional de I+D+i 2013-2016 and Instituto de Salud Carlos III, Subdirección General de Redes y Centros de Investigación Cooperativa, Ministerio de Ciencia, Innovación y Universidades, Spanish Network for Research in Infectious Diseases (REIPI RD16/0016/0001), co-financed by the European Development Regional Fund A way to achieve Europe, Operative Program Intelligence Growth 2014–2020. There was no additional external funding received for this study.

4. Thank you for stating the following financial disclosure:

[The MODERN studies (Understanding and modelling reservoirs, vehicles and transmission of ESBL-producing Enterobacteriaceae in the community and long term care facilities) were part of a Joint Programming Initiative on Antimicrobial Resistance collaborative research project, under the 2016 Joint Call framework (Transnational Research Projects on the Transmission Dynamics of Antibacterial Resistance). It received funding from the following national research agencies: Instituto de Salud Carlos III (grant no. AC16/00076), Netherlands Organization for Health Research and Development (grant no. 681055, 547001004), Swiss National Science Foundation (grant no. 40AR40-173608), German Federal Ministry of Education and Research (grant no. 01KI1701), the French Agence Nationale de la Recherche (grant no. ANR-16-JPEC-0007-03), and UK Medical Research Council (grant no. MR/R004536/1). Elena Salamanca, and Jesús Rodríguez-Baño received support for research from by the Plan Nacional de I+D+i 2013-2016 and Instituto de Salud Carlos III, Subdirección General de Redes y Centros de Investigación Cooperativa, Ministerio de Ciencia, Innovación y Universidades, Spanish Network for Research in Infectious Diseases (REIPI RD16/0016/0001), co-financed by the European Development Regional Fund A way to achieve Europe, Operative Program Intelligence Growth 2014–2020.].

Please state what role the funders took in the study. If the funders had no role, please state: "The funders had no role in study design, data collection and analysis, decision to publish, or preparation of the manuscript."

If this statement is not correct you must amend it as needed.

Please include this amended Role of Funder statement in your cover letter; we will change the online submission form on your behalf.

We have amended ‘Role of Funder statement’
**Role of Funder statement**
The funders had no role in study design, data collection and analysis, decision to publish, or preparation of the manuscript.

5. Thank you for stating the following in your manuscript:

[The MODERN studies (Understanding and modelling reservoirs, vehicles and transmission of ESBL-producing Enterobacteriaceae in the community and long term care facilities) were part of a Joint Programming Initiative on Antimicrobial Resistance collaborative research project, under the 2016 Joint Call framework (Transnational Research Projects on the Transmission Dynamics of Antibacterial Resistance). It received funding from the following national research agencies: Instituto de Salud Carlos III (grant no. AC16/00076), Netherlands Organization for Health Research and Development (grant no. 681055, 547001004), Swiss National Science Foundation (grant no. 40AR40-173608), German Federal Ministry of Education and Research (grant no. 01KI1701), the French Agence Nationale de la Recherche (grant no. ANR-16-JPEC-0007-03), and UK Medical Research Council (grant no. MR/R004536/1). Elena Salamanca, and Jesús Rodríguez-Baño received support for research from by the Plan Nacional de I+D+i 2013-2016 and Instituto de Salud Carlos III, Subdirección General de Redes y Centros de Investigación Cooperativa, Ministerio de Ciencia, Innovación y Universidades, Spanish Network for Research in Infectious Diseases (REIPI RD16/0016/0001), co-financed by the European Development Regional Fund A way to achieve Europe, Operative Program Intelligence Growth 2014–2020.]

We note that you have provided funding information that is currently declared in your Funding Statement. However, funding information should not appear in the Acknowledgments section or other areas of your manuscript. We will only publish funding information present in the Funding Statement section of the online submission form.

Please remove any funding-related text from the manuscript and let us know how you would like to update your Funding Statement. Currently, your Funding Statement reads as follows:

[The MODERN studies (Understanding and modelling reservoirs, vehicles and transmission of ESBL-producing Enterobacteriaceae in the community and long term care facilities) were part of a Joint Programming Initiative on Antimicrobial Resistance collaborative research project, under the 2016 Joint Call framework (Transnational Research Projects on the Transmission Dynamics of Antibacterial Resistance). It received funding from the following national research agencies: Instituto de Salud Carlos III (grant no. AC16/00076), Netherlands Organization for Health Research and Development (grant no. 681055, 547001004), Swiss National Science Foundation (grant no. 40AR40-173608), German Federal Ministry of Education and Research (grant no. 01KI1701), the French Agence Nationale de la Recherche (grant no. ANR-16-JPEC-0007-03), and UK Medical Research Council (grant no. MR/R004536/1). Elena Salamanca, and Jesús Rodríguez-Baño received support for research from by the Plan Nacional de I+D+i 2013-2016 and Instituto de Salud Carlos III, Subdirección General de Redes y Centros de Investigación Cooperativa, Ministerio de Ciencia, Innovación y Universidades, Spanish Network for Research in Infectious Diseases (REIPI RD16/0016/0001), co-financed by the European Development Regional Fund A way to achieve Europe, Operative Program Intelligence Growth 2014–2020.].

Please include your amended statements within your cover letter; we will change the online submission form on your behalf.

We removed the funding statement from the manuscript.

6. Please amend the manuscript submission data (via Edit Submission) to include authors TD Verschuuren, J Guther, ME Riccio, D Martak, E Salamanca, S Göpel, N Conzelmann, J Scharringa, P Musicha, IB Autenrieth, BS Cooper, D Hocquet, E Tacconelli, J Rodriguez-Baño, S Harbarth, AC Fluit, S Peter, and JAJW Kluytmans.

It’s not clear what needs to be amended here, the author list is correct. Should first names be replaced to first letter only? The author list form in the submission data in the editorial manager page seems to request full first names.

7. Please amend your authorship list in your manuscript file to include authors Tess Verschuuren, Julia Guther, Maria Eugenia Riccio, Daniel Martak, Elena Salamanca, Siri Göpel, Nadine Conzelmann, Jelle Scharringa, Patrick Musicha, Ingo B. Autenrieth, Ben S. Cooper, Didier Hocquet, Evelina Tacconelli, Jesus Rodriguez-Baño, Stephan Harbarth, Ad C. Fluit, Silke Peter, and Jan A.J.W. Kluytmans.

We amended authorship list to

Tess D. Verschuuren^1,2^, Julia Guther^3^, Maria Eugenia Riccio^4^, Daniel Martak^5,6^, Elena Salamanca^7^, Siri Göpel^8^, Nadine Conzelmann^8^, Jelle Scharringa^9^, Patrick Musicha^10^, Ingo B. Autenrieth^11^, Ben S. Cooper^10^, Didier Hocquet^5,6^, Evelina Tacconelli^8,12^, Jesús Rodríguez-Baño^7^, Stephan Harbarth^4^, Ad C. Fluit^9^, Silke Peter^3^, Jan A. J. W. Kluytmans^2,9^

8. Please include captions for your Supporting Information files at the end of your manuscript, and update any in-text citations to match accordingly. Please see our Supporting Information guidelines for more information: <http://journals.plos.org/plosone/s/supporting-information>.

A supporting information section was added to the manuscript

If the reviewer comments include a recommendation to cite specific previously published works, please review and evaluate these publications to determine whether they are relevant and should be cited. There is no requirement to cite these works unless the editor has indicated otherwise.

Not applicable

Please review your reference list to ensure that it is complete and correct. If you have cited papers that have been retracted, please include the rationale for doing so in the manuscript text, or remove these references and replace them with relevant current references. Any changes to the reference list should be mentioned in the rebuttal letter that accompanies your revised manuscript. If you need to cite a retracted article, indicate the article’s retracted status in the References list and also include a citation and full reference for the retraction notice.

Done

**Reviewers' comments:**

Reviewer #1: The authors present a study on the epidemiology of extended-spectrum beta-lactamase (ESBL)-producing *E. coli* and *K. pneumoniae*. The work is based on isolates collected from five European metropolitan areas, which were genome-sequenced and analyzed using *in silico* approaches. Analyses of antimicrobial resistance gene (ARG) content, core genome multilocus sequence typing (cgMLST), and core genome comparisons allowed the authors to explore clonal relationships between isolates. The topic is of public health relevance, and the manuscript is generally well-written and easy to follow.

Reviewer 1, thank you for taking the time to review our manuscript.

Minor comments:

The possible role of the hospital or LTCF environment as a reservoir or amplifier of multidrug-resistant bacteria is not discussed. This would be particularly relevant given the healthcare-associated nature of the study population.

We have added the following sentence to the discussion (L265) ‘Hospital and LTCF environments are well-established as significant reservoirs and amplifiers of multi-drug resistant bacteria, contributing to the persistence and spread of these organisms within healthcare settings and the broader community [16, 24, 25].’

The limitations section occupies more than half of the discussion and could be slightly condensed to improve balance with the rest of the text.

Agreed, we have shortened the limitations section to about 2/3rds of the original length.

While the authors mention the need for long-read sequencing, they could elaborate more specifically on the types of questions that such technology would enable (e.g., plasmid structure, transmission dynamics of mobile genetic elements).

Thank you for this suggestion. We find that this point is the main limitation of the study, and future results from nanopore sequencing analyses are of interest to the scientific community.
We have added a few details on challenges with assembly due to repetitive sequences (L272), but refrained from adding more information on long-read sequencing for the sake of brevity. ‘Several limitations must be acknowledged. Firstly, horizontal gene transfer of ARGs could not be assessed due to limitations of short-read sequencing technology in reconstructing mobile genetic elements such as plasmids [26]. Ongoing MODERN studies using previously completed nanopore sequencing aim to address plasmid characterization, overcoming any issues with repetitive sequence elements.’

Would the authors consider routine genomic surveillance in hospitals or LTCFs useful? This could be suggested as a potential recommendation for future practice.

As the consideration of genomic surveillance as a routine procedure would ensuite a more detailed discussion about the appropriateness of the effort and the benefits, and should furthermore incorporate discussion about AMR in mobile genetic elements, we refrain from elaborating such recommendations within this manuscript.

Reviewer #2: The study addresses a very important and pertinent issue. Epidemiological, laboratory and statistical methodology was great and innovative. As far as results are concerned, there was a bit if discrepancy between the text on line 158 and the table where text says Table 1 shows mrc-1 gene in an ESBL-EC strain while text indicates it is in an ESBL- Kp!!! Otherwise the results are well represented and easy to understand. The discussion analyses the results notes the limitations. Overall study is well designed, done and excellently written.

Reviewer 2, thank you for taking the time to read and comment on our MS, many thanks for pointing this discrepancy out. The *mrc-1* gene indeed belonged to an ESBL-Kp from food collected in Tübingen (isolate_625 in S2_Table). Table 2 was incorrect and has been adjusted accordingly.
